# Household Engagement in Both Aquaculture and Horticulture Is Associated with Higher Diet Quality than Either Alone

**DOI:** 10.3390/nu12092705

**Published:** 2020-09-04

**Authors:** Rumana Akter, Nobuyuki Yagi, Hiroaki Sugino, Shakuntala H. Thilsted, Shibani Ghosh, Sabi Gurung, Katherine Heneveld, Robin Shrestha, Patrick Webb

**Affiliations:** 1 Graduate School of Agricultural and Life Sciences, The University of Tokyo, Tokyo 1138657, Japan; yagi@g.ecc.u-tokyo.ac.jp (N.Y.); a-sugino@g.ecc.u-tokyo.ac.jp (H.S.); 2 WorldFish, Jalan Batu Maung, Batu Maung, Bayan, Lepas, Penang 11960, Malaysia; s.thilsted@cgiar.org; 3 Friedman School of Nutrition Science and Policy, Tufts University, Boston, MA 02111, USA; Shibani.Ghosh@tufts.edu (S.G.); sabigrg1@gmail.com (S.G.); Katherine.Heneveld@tufts.edu (K.H.); Robin.Shrestha@tufts.edu (R.S.); Patrick.Webb@tufts.edu (P.W.); 4 Feed the Future Innovation Lab for Nutrition, Tufts University, Boston, MA 02111, USA

**Keywords:** diet quality, micronutrients, aquaculture, horticulture

## Abstract

The consumption of high-quality diverse diets is crucial for optimal growth, health, and wellbeing. Objective: This study assessed the diet quality of households by their type of engagement in homestead aquaculture and/or horticulture. Socio-demographic determinants of diet quality were also studied. Method: Diet quality was assessed using a nutrient adequacy ratio (NAR), based on the preceding 7 days’ dietary recall at the household level. Adult male equivalent units (AMEs) were used for age- and sex-specific intra-household distribution of household intakes. Mean adequacy ratios (MAR) were computed as an overall measure of diet quality, using NAR. Results: Better diet quality (mean ± SD) was associated with households engaged in both homestead aquaculture and horticulture (0.43 ± 0.23; *p* < 0.001) compared to only one type of agriculture (0.38 ± 0.20) or none (0.36 ± 0.20). Tukey’s post-hoc test confirmed significant differences in diet quality between both and either engagement (0.05 ± 0.01, *p* < 0.001), both and no engagement (0.07 ± 0.01, *p* < 0.001), and either and no engagement households (0.02 ± 0.01, *p* < 0.001). Beyond farm production of nutrient-rich foods, generalized estimating equations showed that diet quality was influenced by the higher educational level and occupation of adult household members, higher daily per capita food expenditure, sex, family size and region. Conclusions: Projects that promote and support household engagement in both homestead aquaculture and horticulture have the potential to improve the diet quality of households.

## 1. Introduction

Poor diet quality, commonly measured as a lack of diversity across food groups consumed, is associated with higher nutritional deficiencies globally, and especially in low-income countries [1]. Young children and women are particularly vulnerable to this situation [2,3]. Undernutrition continues to be a serious public health concern in Bangladesh, especially among young children (6–59 months) and women of reproductive age (15–49 years) [4]. The prevalence of overweight and obesity among this group of women is also increasing [5], while micronutrient (essential vitamins and minerals) deficiencies are still widespread across Bangladesh [6,7,8,9]. Common micronutrient deficiencies reported in Bangladesh are vitamin A, iron, calcium, folic acid, zinc, vitamin B12, and iodine [10]. The co-existence of these nutritional problems reflect sub-optimal diets that tend to be high in energy but low in diversity, and particularly low in nutrient-rich foods such as fish, fruits, vegetables, dairy and legumes [10,11,12,13,14,15].

Studies have shown that the diets of smallholder households are often dependent on food supplied from their own production [16,17]. There is some evidence of households’ own horticulture supporting lower micronutrient deficiencies, but evidence of the impact of households’ own livestock production on the intake of key nutrients is mixed depending on the context [18,19,20]. What is more, increased food production at the household level is known to be associated with decreased undernutrition among young children and women, although it is unclear if this relationship is mediated through the direct food consumption pathway [21], via the income pathway or via a combination of both pathways.

One way to increase the intake of nutrient-rich foods in Bangladesh is via increased aquaculture and/or horticulture. Households engaging in such activities may have the potential to improve their diets through direct consumption of nutrient-rich foods (e.g., fish, fruits and vegetables) from their own production and/or indirectly, through purchasing other nutrient-rich diverse foods from the market, through income generated by selling homegrown produce.

This study assesses the diet quality of households in the southwest of Bangladesh, where several development projects have been implemented, including aquaculture and horticulture activities, with the aim of increasing production and improving the nutrition of the household either through the direct and/or indirect pathways of agriculture and nutrition [22]. The aim of the study was to determine whether households actively engaged in both homestead aquaculture and horticulture have better diet quality than those engaged in either one, or households not directly engaged in agriculture. Socio-demographic determinants (educational level and occupation of adult household members, per capita monthly income and daily per capita food expenditure of the household, and women educational level) of diet quality were also studied. This study does not aim to evaluate the impact of any given project or project components; instead, it seeks to understand which types of agricultural practices influence diet quality.

## 2. Materials and Methods

Data for this paper were sourced from the “Bangladesh Aquaculture-Horticulture for Nutrition Research (BAHNR)” study conducted by the Feed the Future Innovation Lab for Nutrition. The BAHNR study collected three rounds of data (at 6-month intervals) as part of a longitudinal observational cohort study. The first round of BAHNR data, collected between January and April 2016, were used for this secondary analysis. The study was conducted in 102 unions (the smallest administrative unit of a subdistrict/division) across three regions (Dhaka, Barisal and Khulna) of southwestern Bangladesh. The study utilized a sampling strategy used previously and rendered a sample that was representative of the United States Agency for International Development (USAID) Feed the Future Zone of Influence [23]. A total of one hundred and two unions were randomly selected from a total of 1115 unions in the three regions. Subsequently, for the purpose of the BAHNR study objectives (unrelated to this paper), these 102 unions were classified into groups by the presence of an exposure (one or more USAID-funded projects or no project implemented in a union) [24]. The study unions were stratified into three groups: group 1, exposed to at least one intervention project (28 unions); group 2, exposed to two or more intervention projects (32 unions); group 3, not exposed to any intervention project (42 unions). In each union, a total of 30 households were randomly selected and followed up on in the three survey rounds.

Household level diet quality was assessed using the preceding seven days’ dietary recall of the household. To calculate household level nutrient intakes, adult male equivalent units (AMEs) were used for the age- and sex-specific intra-household distribution of household intakes. The intake of each nutrient (macro- and micronutrient) was computed for a seven-day period and standardized to obtain a daily intake per household.

The diet quality of the individual was assessed by computing the nutrient adequacy in the diet using the nutrient adequacy ratio (NAR) [25,26]. Mean adequacy ratio (MAR) was computed as an overall measure of diet quality using NAR [25]. Eleven micronutrients: iron, calcium, zinc, vitamin A, thiamine, riboflavin, niacin, vitamin B6, folate, vitamin B12, and vitamin C, in addition to energy, were selected for assessment. These micronutrients reflect key dimensions of diet quality [27], and are considered as the ‘nutrients of concern’ globally [11,28,29].

Ethical approval for the study was obtained from the Bangladesh Medical Research Council, Dhaka, Bangladesh (reference: BMRC/NREC/2013-20161623), as well as the Tufts University Health Sciences Campus Institutional Review Board, in Boston, Massachusetts (IRB# 11954). Prior to enrollment in the study, written consent was obtained from all participants.

### 2.1. Sources of Dietary Data

Dietary data were collected by locally hired, Bengali-speaking, trained enumerators. The enumerators were trained on a Bengali version of the questionnaire which was used for pre-testing prior data collection. Enumerators had a list of 292 foods, and asked participants if the household consumed each food in the past 7 days. If they responded yes, the enumerator asked follow-up questions, including about the quantity of the food item. Data on the source of the acquired foods (i.e., whether food was home produced, purchased, or was a gift from neighbors/relatives) were also collected. The categories of food items used included cereals, pulses, leafy and non-leafy vegetables, fruits, fish, milk, eggs, meat, spices, drinks and beverages, edible oils, and mixed dishes (e.g., fish/meat/egg and vegetables dishes). Foods eaten away from home and the ingredients of purchased foods were also accounted for.

### 2.2. Nutrient Database

The main source of nutrient data for this analysis was the most recent Bangladeshi food composition database [30]. However, since the Bangladeshi food composition database does not have nutrient information for all food items and mixed dishes consumed by study households, a new food composition database was compiled for this study, combining the Indian food composition table [31], the Nepalese food composition table [32], and the United States Development of Agriculture (USDA) national nutrient database for standard reference legacy release, April 2018, to supplement the Bangladesh data [33]. All food items and mixed dishes consumed by households were converted to nutrients using the compiled food composition database.

### 2.3. Intra-Household Food Allocation Using Adult Male Equivalent (AME) Fractions

As household members do not have equitable access to food and/or do not consume the same amounts of food, AME for the age- and sex-specific intra-household distribution of household level dietary intakes was calculated, following the steps outlined by Claro et al. [34]. AMEs were estimated using the mean energy requirements of women and men from 19 to 50 years of age, with moderate physical activity, resulting in a reference value of 2550 kcal per day, as recommended by the national research council [35]. An additional 300 kcal per day was added for pregnant women and 500 kcal per day for lactating women. The AMEs ranged from 0.29 for newborns to 1.18 for men aged 15 to 18 years [34] (Appendix A).

### 2.4. Estimating Daily Nutrient Intakes of Individual

The intake of total household energy and micronutrients (all 11 nutrients) was determined for individual household members, according to the age- and sex-specific AME, in accordance with former studies [36,37]. Subsequently, nutrient intakes were compared with the recommended nutrient intakes of an individual, considering age and sex [38]. Considering the overall composition of diets (mixed diets with fish protein, and unfermented, unrefined cereal grains and flour, high phytate and low ascorbic acid) of rural Bangladeshi people, the cutoffs for the moderate bioavailability of zinc and the 12% bioavailability of iron were used, as suggested by the joint consultation of the Food and Agriculture Organization (FAO) and the World Health Organization (WHO) [38].

### 2.5. Measuring NAR and MAR

NAR for a given nutrient is the ratio of the individual’s intake to the current recommended intake of individual, considering age and sex [26,39]. The NAR values were truncated at 1 so that a nutrient with a NAR greater than 1 could compensate for a nutrient with a lower NAR. MAR was calculated by averaging all truncated NAR values together, as described in Equation 2. Thus, MAR is reported on a scale from 0 to 1, with 0 indicating that the requirement for no nutrients was met, and 1 indicating that the requirements for all nutrients were met [25].

(1) $$\mathrm{NAR}=\frac{\mathrm{Daily}\;\mathrm{nutrient}\;\mathrm{intake}}{\mathrm{Recommended}\;\mathrm{nutrient}\;\mathrm{intake}}$$

(2) $$\mathrm{MAR}=\frac{\sum^{\;}\mathrm{NAR}\;\left(\mathrm{each}\;\mathrm{truncated}\;\mathrm{at}\;1\right)}{\mathrm{Number}\;\mathrm{of}\;\mathrm{nutrients}}$$

### 2.6. Statistical Analysis

Data were analyzed using the statistical software Stata (version 15.1). Descriptive statistics were calculated to describe the daily per capita intake in grams (mean ± standard error (SE)) of each food group and the corresponding nutrient value of food intakes at household and individual levels (mean ± SD). A one-way analysis of variance (ANOVA) was conducted, to assess overall nutrient adequacy (mean MAR) in the diet of households and to test the differences in daily per capita intake (in grams) of major food groups, by the types of household engagement (i.e., aquaculture and/or horticulture production). Tukey’s post-hoc test was conducted to confirm the mean differences in diet quality/MAR between types of households.

Logistic regression models examined the constant effect of aquaculture and/or horticulture engagement on the likelihood of adequate dietary intake of each nutrient. These models were adjusted for the potential influence of educational level and occupation of adult household members, per capita monthly income and daily per capita food expenditures of the household, sex, age, family size, and region.

One-way ANOVA was used to test the differences in daily per capita intake in grams of major food groups, by type of household engagement. Women’s educational level (the mother or caregiver of the selected child) and occupation of adult household members, and daily per capita food expenditures of the household (expressed as quartiles, 1st (lowest), 4th (highest)) influencing the diet quality of household members were also assessed using one-way ANOVA.

Due to the clustered data and repeated responses from the same household, a generalized estimating equation (GEE) model was used to examine the associations of diet quality with relevant individuals (i.e., education and occupation of adult household members, and sex) and household-level factors (e.g., household engagement in aquaculture and/or horticulture, daily per capita food expenditure of the household, family size, and region).

The educational level of women (mother/caregiver of the selected child) was categorized into four categories, namely: primary level, secondary level, college level, and graduate level. The current main occupation of each adult household member (women and men, age >18 years) was categorized into four categories, namely: no earning, day labor, self-employed, and monthly salary/business. Agricultural day laborers, construction labors, cleaners, earth workers, and factory workers who received daily wages were considered as day laborers. Self-employed individuals included tailors, carpenters, potters, cobblers, village doctors, and electricians. Monthly salaried/business people were defined as those either receiving a monthly salary or receiving a certain amount of money on a monthly basis from their job/business.

## 3. Results

### 3.1. Sample Characteristics

About half of the study population (43.3%) was 19–49 years of age (Table 1), followed by ≤5 years (22.3%), ≥50 years (14.3%), 6–12 years (13.8%), and 13–18 years of age (6.2%). More than one third (35.9%) of adult household members had an education up to secondary level, with just over one quarter (27.7%) reporting primary-level education. More than one quarter of adults (27.9%) had no formal education and very few (2.7%) had a graduate-level education.

Farming (working on own farm, sharecropper/tenant farmer, homestead food production of fish/livestock/poultry) was the main occupation for about one third of adult men (34.2%), followed by self-employed (about 18%; rickshaw puller, barber, tailor, village doctor), and wage labor (15.4%; agriculture day labor, construction worker, factory worker). Most adult women (92%) were not involved in any income-related activities.

### 3.2. Diet Quality of Household Members by Type of Household Engagement with Aquaculture and/or Horticulture

Table 2 assessed differences in diet quality in households engaged in both (aquaculture and horticulture), either (aquaculture or horticulture), and no engagement (neither aquaculture nor horticulture), *F* (2, 14,330) = 101.42, *p* < 0.001. Higher diet quality (mean ± SD) was associated with households engaged in both aquaculture and horticulture (0.43 ± 0.23, *p* < 0.001) compared to either (0.38 ± 0.20) or no engagement (0.36 ± 0.20); *F* (2, 14,330) = 101.42, *p* < 0.001.

### 3.3. Pairwise Comparison of Mean MAR with Equal Variance (Tukey’s Post-Hoc Test)

Tukey’s post-hoc test confirmed that diet quality was significantly higher (*p* < 0.001) in households engaged in both activities compared to either or no type of engagement (Table 3). Similarly, diet quality was significantly higher (*p* < 0.01) in households engaged in either aquaculture or horticulture compared to no engagement.

### 3.4. Diet Quality of Households by Type of Household Engagement with Aquaculture and/or Horticulture

Logistic regression models (Table 4) showed that meeting dietary micronutrient and energy intake recommendations were significantly and positively associated (*p* < 0.01) with households engaged in both types compared to either or no type of engagement, except for vitamin B6 and vitamin C (*p* > 0.05). Furthermore, calcium intake was strongly and positively associated with households engaged in both types of value-added agriculture (odds ratio (OR) = 2.42, *p* < 001, *B* = 0.88). Riboflavin (OR = 1.81, *B* = 0.59, *p* < 0.001), niacin (OR = 1.70, *B* = 0.53, *p* < 0.001), folic acid (OR = 1.66, *B* = 0.50, *p* < 0.001), iron (OR = 1.56, *B* = 0.44, *p* < 0.001), and thiamin (OR = 1.52, *B* = 0.42, *p* < 0.001) intakes were moderately associated with both forms of agricultural activity. Meeting dietary micronutrient and energy intake recommendations were significantly and positively associated (*p* < 0.01) with households engaged in either type compared to no engagement, except for vitamin B6 (*p* > 0.05). All models were adjusted for the education and occupation of household members, per capita monthly income and the daily per capita food expenditure of the household, sex, age, family size, and region.

### 3.5. Quantity of Intake (g/Person/Day) of Major Food Groups by Household Types

**Figure 1:**
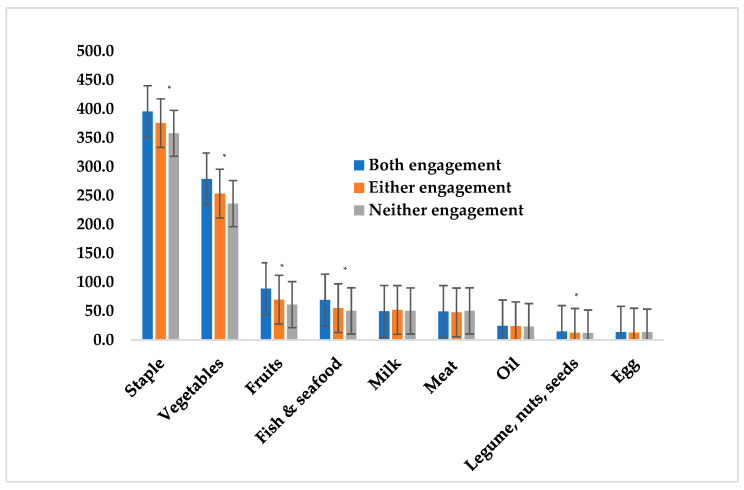
Quantity of intake (g/person/day) of major food groups by household types. * *p* < 0.01; fish & seafood: all types; meat: beef, lamb and mutton, sheep, goat, and pork (all types).

Figure 1 assesses the daily per capita quantity of intake by food groups, by type of farming engagement. Quantities of fish & seafood, vegetables, fruits, legumes/nuts/seeds and staple food intakes were significantly higher (278.8 g/person/d; 88.9 g/person/d; 69.0 g/person/d; 14.7 g/person/d; and 395.4 g/person/d respectively; *p* < 0.001) in households engaged in both aquaculture and horticulture compared to either or none. The source of fish & seafood and vegetables consumed in households with both or either engagement was mostly from households’ own production.

### 3.6. Socio-Demographic Determinants of Diet Quality of Household Members

Significant differences in mean MAR among women with differing levels of education (*F* (3, 3007) = 48.55, *p* < 0.001) were found (Table 5). Women with a higher educational level had a higher MAR (mean ± SD) (graduate level, 0.47 ± 0.18) compared to women with a lower educational level (college level, 0.43 ± 0.19; secondary level, 0.36 ± 17; and primary level, 0.30 ± 0.17), and the difference was significant.

There were significant differences in MAR among household members of different occupation categories, determined by one-way ANOVA (*F* (3, 8261) = 153.81, *p* < 0.001). The mean MAR was higher among household members (mean ± SD) who had a monthly salaried job or business (0.47 ± 0.21) compared to other occupation categories (self-employed, 0.42 ± 0.21; day laborer, 0.36 ± 0.19; and no earning, 0.34 ± 0.18).

The daily per capita food expenditure of the household was categorized into four quartiles (Table 5). A higher MAR (mean ± SD) of any household member was associated with a higher per capita food expenditure quartile (fourth quartile, 0.53 ± 0.21) compared to the lower food expenditure quartiles (third quartile, 0.42 ± 0.19; second quartile, 0.34 ± 0.18; and first quartile, 0.28 ± 0.18), tested using one-way ANOVA (*F* (3, 14,329) = 1159.06, *p* < 0.001).

### 3.7. Generalized Estimating Equation Predicted Overall Diet Quality of Households

A weighted generalized estimating equation (GEE) was used to predict the overall diet quality of households (keeping MAR or diet quality as the dependent variable) (Table 6). The model was adjusted for the educational level and occupation of adult household members, daily per capita household food expenditure, family size, sex, and region. The overall model is significant (*B* = 0.41, *p* < 0.001). Diet quality was significantly better (*p* < 0.001) in households engaged with both aquaculture and horticulture. Furthermore, diet quality was significantly better (*B* = −0.05, *p* < 0.001) in households with either form of agriculture compared to no engagement (*B* = −0.08, *p* < 0.001). Daily per capita household food expenditure was significantly associated (*B* = 0.004, *p* < 0.001) with the diet quality of a household, although the gradient was small. Households with an adult member who had a monthly salaried job or business had a significantly better diet quality compared to household members in a household with no income earner or self-employed (*B* = 0.03, *p* < 0.001) or day wage laborers (*B* = 0.04, *p* < 0.001). Adult household members with formal education up to graduate or college level had a significantly better diet quality compared to those with education up to secondary (*B* = −0.02, *p* < 0.05) or primary level (*B* = −0.05, *p* < 0.001). Sex of household member, family size, and geographical location also influenced diet quality of household members.

## 4. Discussion

Diet quality, as measured using the MAR, was significantly higher in households that were engaged in both homestead aquaculture and horticulture (*p* < 0.001). A similar result was found between households actively producing either form of nutrient-rich foods versus none. There are several plausible explanations for these relationships. Firstly, we know of several intervention projects that have been implemented in the survey areas. These projects focused on improving the production and productivity of aquaculture and/or horticulture by increasing access to improved inputs alongside social and behavior change communication (SBCC) supporting the increased intake of nutrient-rich foods. Secondly, households that produced nutrient-rich foods were likely to consume these same foods. These findings are consistent with those from other studies that have demonstrated that people eat many of the foods they produce [40,41,42,43,44]. Dietary intakes largely depend on foods supplied from households’ own farms among smallholders, particularly if they are more distant from food markets [16,17,45]. Women and men in households engaged in aquaculture and/or horticulture received technical training from extension agents on improved production technologies; men were more receptive to their wives’ knowledge and abilities and sought their advice and input, while also collaborating together through sharing workload in the field and at home [46]. Furthermore, women in these households also received nutrition training (nutrition education) on the importance of eating diverse, nutritious foods, which might have also contributed to better diet quality among members in households.

Logistic regression analysis showed that dietary micronutrient and energy intakes were significantly (*p* < 0.01) and positively associated with aquaculture and horticulture combined, except for vitamin B6 and vitamin C. Dietary calcium intake was strongly correlated with households engaged in aquaculture and horticulture, followed by riboflavin, niacin, and folic acid. Fish farming in Bangladesh covers a range of variants; that is, it does not only mean the production of a single species aimed at maximizing productivity. For example, this region of Bangladesh has households farming both micronutrient-rich ‘small fish’ and larger fish species in the same ponds (e.g., a combination of mola and carp species and/or tilapia and shrimp). The small fish species mola is an exceptionally rich source of multiple micronutrients such as calcium, iron, zinc, riboflavin, folic acid, vitamin A, and vitamin B12. Similarly, horticulture is not framed around a single fruit or vegetable, with some households focusing on tomatoes and onions, but others including orange sweet potato (OSP) and other products for home consumption as well as sale in the market [47,48]. OSP is a rich source of vitamin A, a micronutrient which is deficient in Bangladeshi diets, particularly the diet of young children (aged 6–59 months) and reproductive-aged women in rural Bangladesh [6,30,49,50,51].

These findings are consistent with studies that have demonstrated that agricultural interventions seeking to increase productivity of specific nutrient-rich foods, coupled with nutrition education, can be positively associated with an increased intake of targeted foods [45,52,53]. These findings are further reinforced by the results showing that the quantities of daily per capita intake of fish & seafood, vegetables, and fruits were significantly higher in households engaged in both forms of value-added agriculture versus none.

As reported by households with both forms of engagement, the source of nutrient-rich foods was mostly from households’ own production. Fish is the most commonly consumed animal source food (ASF) in Bangladesh [43,54,55] compared to other ASFs such as chicken, milk, eggs, duck, and red meat. Studies have shown that adding a small amount of ASFs to a plant-based diet can enhance the absorption of vitamins and minerals from these foods, and can significantly impact maternal health and child development [1,10,13]. The availability and accessibility of micronutrient-rich foods from households’ own production, combined with social and behavior change messaging, through individual interaction and/or group meetings or mass media, probably led to increased dietary nutrient intakes in households engaged in both aquaculture and horticulture [20,49,51,56]. Positive associations between intakes of other dietary nutrients (iron, thiamine, vitamin A, vitamin B12, and zinc) and households that practiced both aquaculture and horticulture may also be explained in a similar manner.

Significantly higher quantities of fruits and legumes/nuts/seeds were consumed in households engaged in both or either form of agriculture compared to no engagement. This was likely through the use of their income from the sales of fish and horticulture products, which generates income to purchase other foods. Small, indigenous fish species like mola, which reproduce in a pond, require partial and frequent harvesting, which encourage households to eat them frequently, but large carp species and tilapia are stocked as fingerlings, do not reproduce in the pond and are harvested as adults (after 6–8 months) and sold to generate income, some of which is used to purchase other foods [45]. These results concur with studies conducted in Bangladesh and Cambodia, showing that integrated aquaculture–horticulture is positively associated with farm productivity and household income, which can be used to purchase additional foods for the household [6,57,58,59].

Women’s educational level, the occupation of adult household members, and daily per capita household food expenditure were important determinants of diet quality, as predicted by one-way ANOVA. These findings are consistent with those of other studies in South Asia, including Bangladesh, demonstrating the positive impact of women’s education, the occupation of adult household members, and the food expenditure of the household in terms of the diet diversity and quality of household members [17,60,61,62]. In women, a higher educational level was associated with a better-quality diet compared to a lower education level. These results are consistent with those from studies demonstrating that women’s education strongly influences the diet quality of household members [61,63,64]. The effect of women’s education on diet quality is stronger than that of the effect of men’s education, as reported in a study in Bangladesh [65]. Women with higher education might have better opportunities to learn and be aware about nutritional knowledge as well to put this knowledge into practice [63].

Better diet quality was also associated with adult household members having a monthly salaried job or regular monthly income from business compared to family members having other occupation categories (no regular earning, day wage labor, or self-employed). Adult household members who had a regular monthly income, either from salaried jobs or business, may have had higher food purchasing capacity, associated with a higher educational level and higher household food expenditure compared to household members in other occupation categories. Similar reasoning may apply to households with a self-employed household member compared to a day wage laborer or someone without a wage income. These results concur with those from studies showing that the employment status and educational level of the adult household members are associated with a greater demand for diversified diets [65,66].

Nonetheless, the better diet quality of the household was associated with a higher daily per capita food expenditure quartile compared to a lower food expenditure quartile. These findings are consistent with results from studies showing that food expenditure was associated with dietary diversity and the nutritional status of household members [16,67]. Increased food expenditure coupled with demographic characteristics and lifestyle play an important role in dietary diversity [67], which is also consistent with the determinants of diet quality in this study.

Using the GEE model, diet quality was associated with households engaged in both aquaculture and horticulture, the higher educational level of adult household members, with members having a salaried job or business, the daily per capita food expenditure of the household, a smaller family size, an adult male and the Dhaka region. Households with more adult male members may have more earning opportunities, which might have influenced the diet quality of the household. Agriculture was the main occupation of most adult male household members in this study. The potential of agricultural development to improve food and nutrition security through food system approaches to provide diverse, nutritious foods has been well documented in a number of studies [19,68,69].

## 5. Conclusions

Household engagement in both aquaculture and horticulture was associated with a better-quality diet compared to either or no engagement. The education and occupation of adults and daily per capita food expenditure were important additional determinants of diet quality.

## Figures and Tables

**Table 1:** Characteristics of studied households and individuals.

	%	*n*
Age distribution (years)		
≤5	22.3	
6–12	13.8	
13–18	06.3	14,333
19–49	43.3	
≥50	14.3	
Sex		
Female	54.7	14,333
Male	45.3	
Educational level of household members (age > 18 years)		
Never attended school	27.9	
Non-formal education	1.2	
Primary	27.7	
Secondary	35.9	
College	4.6	8265
Graduate	2.7	
Occupation of men (age > 18 years)		
Farming	34.2	
Self-employed	18.1	
Trader	16.2	3616
Wage labor	15.4	
No earning	9.5	
Salaried work	6.0	
Production	0.5	
Family size (mean ± ^a^ SD)	4.8 (1.7)	3167

^a^ Standard deviation (SD).

**Table 2:** Diet quality of household members by household engagement in pond aquaculture and/or horticulture (one-way ANOVA).

Types of Household Engagement	Mean ^a^ (MAR)	^b^ SD	*p* Value	*n*
Both engagement	0.43	0.23	<0.001	4449
Either engagement	0.38	0.20		8432
No engagement	0.36	0.20		1452
Total	0.39	0.21		14,333

^a^ Mean adequacy ratio (MAR); ^b^ standard deviation (SD).

**Table 3:** Pairwise comparison of mean MAR with equal variance (Tukey’s post-hoc test).

			Tukey Post-Hoc		
Household engagement	Contrast	^a^ SE	t	*p* value	^b^ 95% CI
Both vs. Either engagement	0.05	0.01	12.67	<0.001	0.040, 0.059
Both vs. No engagement	0.07	0.01	11.04	<0.001	0.056, 0.085
Either vs. No engagement	0.02	0.01	3.48	<0.01	0.007, 0.035

^a^ Standard error (SE); ^b^ confidence interval (CI).

**Table 4:** Associations of dietary nutrient intakes with households engaged in pond aquaculture and/or horticulture (logistic odds ratio).

	Engaged In					
	Aquaculture and Horticulture			Aquaculture or Horticulture		
Nutrients	^a^ OR	^b^ 95% CI	*p* value	^a^ OR	^b^ 95% CI	*p* value
Energy	1.76	1.61, 1.93	<0.001	0.62	0.57, 0.68	<0.001
Calcium	2.42	1.92, 3.04	<0.001	0.47	0.38, 0.59	<0.001
Iron	1.56	1.42, 1.70	<0.001	0.79	0.73, 0.86	<0.001
Zinc	1.22	1.09, 1.37	<0.001	0.88	0.80, 0.97	<0.05
Vitamin A	1.25	1.16, 1.35	<0.001	0.84	0.79, 0.91	<0.001
Thiamin	1.52	1.40, 1.64	<0.001	0.81	0.76, 0.88	<0.001
Riboflavin	1.81	1.61, 2.04	<0.001	0.64	0.57, 0.72	<0.001
Niacin	1.70	1.56, 1.86	<0.001	0.77	0.72, 0.83	<0.001
Vitamin B6	1.08	0.99, 1.19	>0.05	1.00	0.93, 1.08	>0.05
Folic acid	1.66	1.39, 1.98	<0.001	0.71	0.60, 0.84	<0.001
Vitamin C	1.10	0.98, 1.24	>0.05	0.88	0.79, 0.98	<0.05
Vitamin B12	1.31	1.20, 1.42	<0.001	0.82	0.76, 0.88	<0.001

^a^ Odds ratio (OR); ^b^ confidence interval (CI).

**Table 5:** Socio-demographic determinants of diet quality of household members.

Independent Variables	^a^ Mean (MAR)	^b^ SD	*p* Value	*n*
Educational level of women				
Primary	0.30	0.17	<0.001	3011
Secondary	0.36	0.17		
College	0.43	0.19		
Graduate	0.47	0.18		
Occupation category of household members age >18 years				
No earning	0.34	0.18	<0.001	8265
Daily labor	0.36	0.19		
Self-employed	0.42	0.21		
Monthly salary/business	0.47	0.21		
Per capita daily food expenditure of the household				
1st quartile	0.28	0.18	<0.001	14,333
2nd quartile	0.34	0.18		
3rd quartile	0.42	0.19		
4th quartile	0.53	0.21		

^a^ Mean adequacy ratio (MAR); ^b^ standard deviation (SD).

**Table 6:** Generalized estimating equation predicted overall diet quality of households.

	^a^ *B*	^b^ 95% CI	*p* Value
Intercept	0.41	0.38, 0.44	<0.001
Neither engagement	−0.08	−0.10, −0.06	<0.001
Either engagement	−0.05	−0.06, −0.03	<0.001
Both engagement	Reference		
Per capita daily food expenditure	0.00	0.00, 0.00	<0.001
Salaried job or business	0.05	0.04, 0.06	<0.001
Daily wage labor	0.04	0.03, 0.05	<0.001
Self-employed	0.03	0.02, 0.04	<0.001
No earning	Reference		
Primary educational level	−0.05	−0.06, −0.04	<0.001
Secondary educational level	−0.02	−0.03, −0.01	<0.05
College educational level	0.00	−0.01, 0.02	>0.05
Graduate educational level	Reference		
Household size	−0.01	−0.01, 0.00	<0.001
Female	−0.06	−0.06, −0.05	<0.001
Male	Reference		
Barisal ^c^	−0.04	−0.05, −0.02	<0.001
Khulna ^c^	−0.03	−0.04, −0.02	<0.001
Dhaka ^c^	Reference		

^a^ Unstandardized beta (*B*); ^b^ confidence interval (CI); ^c^ name of region/administrative division.

## Appendix A

**Table A1:** Adult equivalent conversion factors for estimated energy requirements and distribution of nutrient intakes according to age and sex.

Age (Years)	Energy (Kcal/Day) ^a^	Adult Equivalent Conversion Factors
Infant		
0–1	750	0.29
Children		
1–3	1300	0.51
4–6	1800	0.71
7–10	2000	0.78
Men		
11–14	2500	0.98
15–18	3000	1.18
19–24 ^b^	2900	1.14
25–50 ^b^	2900	1.14
51 +	2300	0.9
Women		
11–14	2200	0.86
15–18	2200	0.86
19–24 ^b^	2200	0.86
25–50 ^b^	2200	0.86
51 +	1900	0.75
Breastfeeding women (+500 kcal/day) ^c^		
11–14	2700	1.06
15–18	2700	1.06
19–24	2700	1.06
25–50	2700	1.06
51 +	2400	0.94
Pregnant women (+300 kcal/day) ^d^		
11–14	2500	0.98
15–18	2500	0.98
19–24	2500	0.98
25–50	2500	0.98
51 +	2100	0.82

^a^ According to Recommended Dietary Allowances (RDA) [37]. ^b^ Age groups used as reference for establishing an adult’s mean energy requirements [36]. ^c^ Additional 500 kcal/day required for breastfeeding women [27]. ^d^ Additional 300 kcal/day required for pregnant women [27]. + Age ≥ 51 years.
